# Field Evaluation of Deltamethrin and Ivermectin Applications to Cattle on *Culicoides* Host-Alighting, Blood-Feeding, and Emergence

**DOI:** 10.3390/v11080731

**Published:** 2019-08-08

**Authors:** Archie K. Murchie, Geoff M. Thompson, Sam Clawson, Andrew Brown, Alan W. Gordon, Stephen Jess

**Affiliations:** 1 Agri-Food and Biosciences Institute, Newforge Lane, Belfast BT9 5PX, Northern Ireland, UK; 2 Ulster Farmers’ Union, 475 Antrim Road, Belfast BT15 3DA, Northern Ireland, UK; 3 Agri-Food and Biosciences Institute, Large Park, Hillsborough BT26 6DR, Northern Ireland, UK

**Keywords:** *Culicoides*, bluetongue, deltamethrin, ivermectin, sticky trapping, onward transmission

## Abstract

The impact of topical applications of deltamethrin and ivermectin to cattle on *Culicoides* spp. landing and blood-feeding was studied in this work using sticky traps mounted on Friesian heifers’ backs. There was no effect of the insecticides on total numbers of *Culicoides* trapped or the proportion engorged. Deltamethrin and ivermectin treatment did not prevent blood-feeding on these animals. Deltamethrin did result in significant *Culicoides* mortality as evidenced by the numbers of dead midges combed from heifers’ upper flanks. The proximity of engorged midges on traps to dead midges in the hair suggests that blood-feeding took place despite midges receiving an ultimately lethal dose of deltamethrin. Ivermectin application resulted in a smaller proportion of nulliparous than parous females caught. There was no significant effect of ivermectin on the numbers of *Culicoides* that emerged from dung samples (but *p* was small at 0.095 for the Obsoletus group *Culicoides*). In cases of suspect animal imports, pour-on or spray applications of deltamethrin could reduce the risk of onward transmission of bluetongue virus.

## 1. Introduction

Biting midges, *Culicoides* spp. (Diptera: Ceratopogonidae), are important vectors of viral pathogens of livestock. During 2006–2009, there was a serious outbreak of the midge-vectored virus, bluetongue virus (BTV) serotype 8, in north-western Europe. The virus causes a notifiable disease and the outbreak resulted in widespread disruption to the cattle and sheep livestock industries. Whilst vaccination was the ultimate solution to the bluetongue outbreak, at early stages of a disease outbreak vaccines may not be developed or available in sufficient quantities for disease management. At these crucial early stages, vector control is a valuable option to prevent spread of the disease [1]. The island of Ireland has remained free from bluetongue, and did so even during the 2006–2009 European outbreak. The main risk of incursion has been from imported livestock [2]. For example, in November 2018, four heifers and one bull were imported into a Northern Irish farm from France. Routine post-import testing found that one of the heifers tested PCR positive for BTV-8 [3]. The infected heifer was euthanized and the other animals quarantined. This was during the ‘vector-free’ period as defined by fewer than five parous *Culicoides* per light trap catch [4], but small numbers of midges were still flying. In cases such as this, insecticide application could reduce the likelihood of onward transmission of the pathogen.

Application of insecticides directly to animals is a common method of nuisance fly management. Two of the most commonly used insecticide groups approved for nuisance fly and ectoparasite control are the synthetic pyrethroids and avermectins (macrocyclic lactones), which both can be conveniently applied by farmers as topical applications, termed ‘pour-ons’, to the skin of the animal. The avermectins can also be applied by sub-cutaneous injections and for internal parasite control as oral doses. Several papers have reviewed *Culicoides* control with respect to disease management, including the use of synthetic pyrethroids and avermectins [1,5,6,7,8].

Whilst there has been much laboratory-based research showing the toxicity of pyrethroids to *Culicoides*, in a review of knowledge gaps in *Culicoides* control, Mullens et al. [9] commented that: “*Protection studies are far more accurate and realistic when we use bait animals and measure natural midge attack and successful engorgement*”. Such studies have mainly used enclosure trapping or drop nets, with midges vacuumed directly from the animals or collected with light traps. Worldwide, the results have been mixed. The most successful trials have been on cattle in Australia, where numbers of *Culicoides* spp. were significantly reduced on treated animals compared to controls—for example, deltamethrin and cypermethrin versus *Culicoides brevitarsis* Kieffer, 1917 [10]; deltamethrin, permethrin, and fenvalerate versus *Culicoides actoni* Smith, 1929 and *Culicoides peregrinus* Kieffer, 1910 [11,12]; and ‘Flyaway’ (a blend of repellents and permethrin) and fenvalerate versus *C. brevitarsis* and *Culicoides wadai* Kitaoka, 1980 [13]. In the US, there was no effect of a dorsal application of a permethrin pour-on on the numbers of *Culicoides sonorensis* Wirth and Jones, 1957 blood-feeding but a belly-spray of permethrin to calves reduced the number of engorged females trapped [14]. In Germany, a deltamethrin pour-on to Holstein-Friesian bulls did not reduce the numbers of engorged Obsoletus and Pulicaris group *Culicoides* caught in light traps [15]. For sheep, a study in Spain found that deltamethrin applied to susceptible areas by hand resulted in zero *Culicoides* engorgement [16] and in Germany a deltamethrin pour-on reduced the numbers of both unfed and blood-fed *Culicoides* collected from drop nets around sheep [17]. Yet in India, dipping sheep in a deltamethrin solution had no impact on *Culicoides* spp. caught in light traps in their pens [18]. Lastly, in the UK a pour-on application of deltamethrin to horses had no effect on light trap catches of Obsoletus and Pulicaris group *Culicoides* from within mesh enclosures [19].

Compared to the pyrethroids, there is less information on the effects of avermectins on *Culicoides*. In Australia, Hereford cattle were given a single subcutaneous injection of ivermectin and *C. brevitarsis* placed in feeding pots on the hosts’ ears. There was no significant effect on feeding propensity but 99% of engorged *C. brevitarsis* died after blood-feeding [20]. However, a comparable experiment in the US, caging *Culicoides variipennis* (Coquillett, 1901) on the shaved skin of ivermectin-injected beef calves, found no significant effect of the same 200 µg per kg of body weight dose on midge mortality [21], probably because the serum concentration was not high enough [22]. In a laboratory bioassay, *C. sonorensis* showed no significant mortality when fed blood from ivermectin-treated horses, sheep or elk, although the infection rate of treated midges with BTV-17 was significantly lower than untreated controls [23]. As avermectins are excreted in the dung, they can also have an effect on *Culicoides* larval survival. In a report on the Australian bluetongue control strategy, it was stated that dung treated with ivermectin was larvicidal for up to 28 days, but no details were given [24].

Thompson et al. [25] used sticky traps to assess on-animal alighting and host preferences between sheep and cattle. The advantage of these sticky trap plates was that they presented a standardised landing area for midges and therefore allowed a comparison between different treatments. They also permitted the animals to move naturally around pasture. However, it is acknowledged that the technique has some limitations. The main limitation is that the sticky plates have to be mounted on the heifers’ mid-backs, or else they will lick the trap or swat it with their tails ruining the catch. Other studies have shown that midges show a predilection for blood-feeding on the lower flanks, underbelly and inner legs where the softer skin and thinner hair may make blood uptake easier [26,27,28].

In this study, the sticky trap method was used in a series of three trials to assess the effects of deltamethrin and ivermectin pour-on applications on *Culicoides* landing and blood-feeding on Friesian heifers in a Northern Ireland pasture. Estimates of the persistence of chemical controls against *Culicoides* vary in the literature. For example, for deltamethrin, Weiher et al. [17] found negative effects on midges 35 days after treatment, whilst Venail et al. [29] predicted maximum mortality after 4 days but that the lethal effect could be as brief as 10 days. For ivermectin, maximum blood concentration occurs 3–4 days after topical application [30,31]. However, in a study on horses the concentration of ivermectin in hair at the pour-on application site was well above that likely to cause *Culicoides* mortality (i.e., 350 ng per mL [22]) at greater than 10 µg per g for at least 40 days [31]. Therefore, the first trial in this study looked at trapping midges for a prolonged period (5 weeks) post application, whilst the following two trials concentrated on a two-week period post application when effects on midges were most likely. In the main, two hypotheses were tested in these trials. The first was that treatment with insecticidal compounds would prevent midge alighting (repellency). The second was that treatment would prevent blood-feeding. In addition, dung samples were taken to assess the effects of drug residues on *Culicoides* emergence from dung.

## 2. Material and Methods

Studies were conducted at the Agri-Food and Biosciences Institute’s (AFBI) Hillsborough research farm, Co. Down, Northern Ireland (54.445290° N, 6.065526° W). The experiments were conducted May–July 2012 (Trial 1) and September 2014 (Trial 2 and 3).

### 2.1. Trial 1—Five Week Study on the Effects of Deltamethrin and Ivermectin on Culicoides’ Landing and Emergence from Dung

The first trial used ivermectin (as Ivomec Classic Pour-On for Cattle, Boehringer Ingelheim Animal Health UK Ltd., Bracknell, UK) and deltamethrin (as Fly and Lice Spot On Insecticide, Zoetis UK Ltd., London, UK). Fifteen Friesian heifers (live weight ~370 kg) were selected from the farm’s herd at random and were allocated to three treatments: five were used as a control, five were treated with ivermectin, and five were treated with deltamethrin one day prior to the start of *Culicoides* monitoring. Animals were treated according to the manufacturers’ instructions. In brief, for ivermectin 1 mL product per 10 kg bodyweight (500 μg ivermectin per kg bodyweight) was applied along the mid-line of the back in a narrow strip between the withers and tailhead. For the deltamethrin, a standard 10 mL of product irrespective of animal weight (0.1 g deltamethrin) was applied as a single dose on the mid-line of the back, at the shoulders.

**Figure 1:**
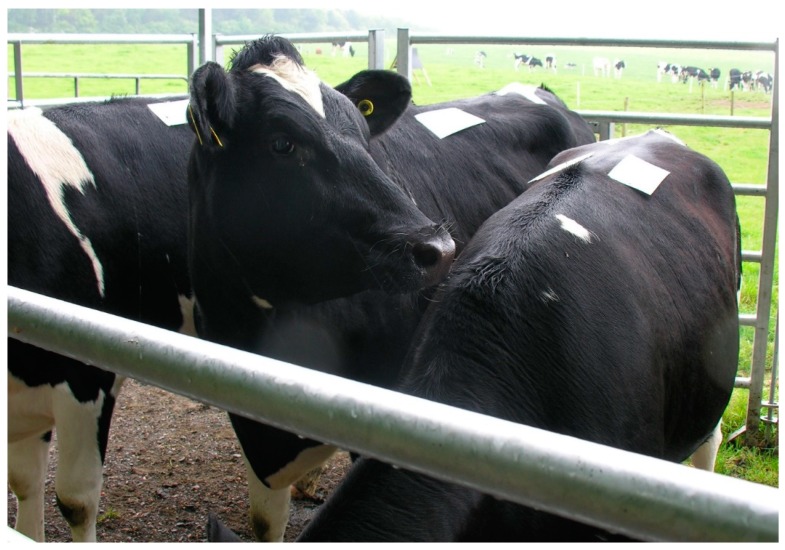
Friesian heifers with two sticky traps mounted mid-back to monitor *Culicoides* landing and blood-feeding.

To monitor *Culicoides* landing, each animal had two white 200 cm^2^ sticky traps (Oecos Insect Monitoring, Kimpton, UK; Agralan Ltd, Swindon, UK) attached to its back using Velcro^®^ and Kamar^®^ adhesive glue (www.kamarinc.com) as per the method of Thompson et al. [25] (Figure 1). Traps were placed on the heifers 1 day after treatment. These traps were left in place for 24 h before being removed. This sampling was repeated at weekly intervals for a total of 5 weeks, equivalent to a period of 30 days after treatment. This first trial examined the persistence of any treatment effects over 5 weeks, whereas the two subsequent trials concentrated on multiple sampling within 15 days of application when insecticides were most bioavailable and effects most likely observed.

*Culicoides* caught on the sticky traps were left in situ. For *Culicoides* identification, specimens were not identified to species but were grouped primarily according to sub-genus and then sub-divided again by morphological characteristics, mainly wing patterning. This approach has been commonly used in the veterinary and applied studies of *Culicoides* [32,33]. The groups were categorised as Obsoletus (*Culicoides chiopterus* (Meigen, 1830); *Culicoides dewulfi* Goetghebuer, 1935; *Culicoides obsoletus* sensu stricto (Meigen, 1919); and *Culicoides scoticus* Downes and Kettle, 1952), Pulicaris (*Culicoides pulicaris* (Linnaeus, 1758); *Culicoides punctatus* (Meigen, 1804); and *Culicoides newsteadi* Austen, 1921), Impunctatus (*Culicoides impunctatus* Goetghebuer, 1920 and *Culicoides grisescens* Edwards, 1939) and Nubeculosus (*Culicoides nubeculosus* (Meigen, 1830), *Culicoides puncticollis* (Becker, 1903), and *Culicoides riethi* Kieffer, 1914) according to the key of Boorman [34]. Additionally, female parity was assessed by abdominal pigmentation using the method described by Dyce [35].

Dung samples were taken from each of the animals, either by collecting from dung pats immediately after defecation or directly from the rectum. Dung samples (300 mL) were laid out in an adjacent paddock in a randomised block design with 1 m between treatments and left exposed to *Culicoides* oviposition for one week before being covered with a bucket emergence trap for 4 weeks to allow for emergence [36]. Following this four week period, the bucket traps were removed and *Culicoides* counted. A soil core (diameter 10 cm and depth 6 cm) was then taken from each dung sample and put into a breathable tissue-culture bag (450 × 200 × 120 mm, Sigma-Aldrich) containing a 100 cm^2^ sticky trap and incubated for a further four weeks at 20 °C (light regime 16 h light, 8 h dark) to determine any midge emergence. Any *Culicoides* caught on the sticky traps were identified to group level and counted. The first dung samples were taken one day after treatment, with sampling repeated at weekly intervals for a total of 5 weeks, resulting in 75 dung samples in total (3 treatments × 5 replicates × 5 weeks); however, the initial samples were destroyed after cattle gained access to the paddock and trampled the emergence traps.

### 2.2. Trial 2—Culicoides’ Landing and Blood-Feeding on Friesian Heifers Treated with Ivermectin Pour-On

Twenty Friesian heifers (live weight ~350 kg) were treated with two antihelmintics. Ten were treated with ivermectin (as Ivomec) as per trial 1, and the other 10 with fenbendazole. The latter treatment equates to the control as animal husbandry practices on the farm did not allow an untreated control at this time but required the use of an antihelmintic against gastrointestinal parasites. Fenbendazole, as Panacur^®^ (MSD Animal Health, Walton, UK), was applied as a 10% oral suspension at 1 mL of the product per 13 kg bodyweight (7.5 mg fenbendazole per kg bodyweight). Five days after treatment, two white sticky traps (200 cm^2^) were mounted to the heifers’ backs for a 24 h period, a process which was repeated again the following day (Table 1). Total numbers of *Culicoides* and those blood-fed were counted immediately after collection.

### 2.3. Trial 3—Culicoides’ Landing, Blood-Feeding and Mortality on Friesian Heifers Treated with Deltamethrin and Ivermectin Pour-Ons

**Figure 2:**
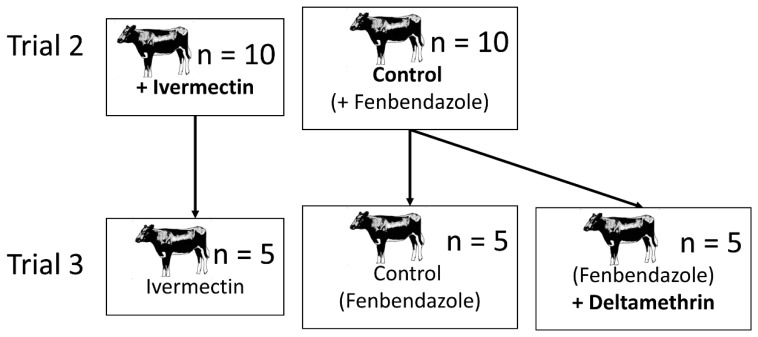
The relationship between the treatments in trial 2 and trial 3. Five heifers selected at random from the trial 2 ivermectin treatment were kept on for trial 3. The 10 heifers in the trial 2 control treatment were split into two groups of five for trial 3. One group was left as a control, whilst the other group was treated with deltamethrin.

Following on directly from trial 2, one week after the initial treatments of ivermectin and fenbendazole, five of the fenbendazole (control) heifers were treated with deltamethrin (as ‘Fly and Lice Spot On Insecticide’) to give an experiment comprising three treatments, i.e., deltamethrin (plus fenbendazole), ivermectin and fenbendazole alone, with five replicate animals (Figure 2). Four days after deltamethrin application, sticky traps were mounted on the backs of heifers for four consecutive 24 h periods (Table 1). In addition to sticky trap sampling, to collect dead *Culicoides* in the heifers’ hair, the upper half of the flanks of each heifer were combed from the withers to hips with a plastic head lice comb (teeth 14 mm length, ~0.3 mm apart) (Superdrug, London, UK) on three consecutive days (Table 1). Combing was done by the same operator each time, taking care to ensure that the areas combed were comparable between animals.

### 2.4. Analyses

*Culicoides* count data were subjected to generalized linear mixed models (GLMMs) fitted with Poisson distributions and logarithmic link functions. Where proportion data were analysed, binomial distributions coupled with logit link functions were used. In the GLMMs, individual animals were modelled as random effects. The significance of the fixed effects in the models was assessed by comparing Wald statistics for each term against an appropriate F-distribution. All analyses were conducted using the statistical package GenStat v16.2 (VSN International Ltd, UK; www.vsni.co.uk).

## 3. Results

### 3.1. Trial 1—Five Week Study on the Effects of Ivermectin and Deltamethrin on Culicoides’ Landing and Emergence from Dung

**Figure 3:**
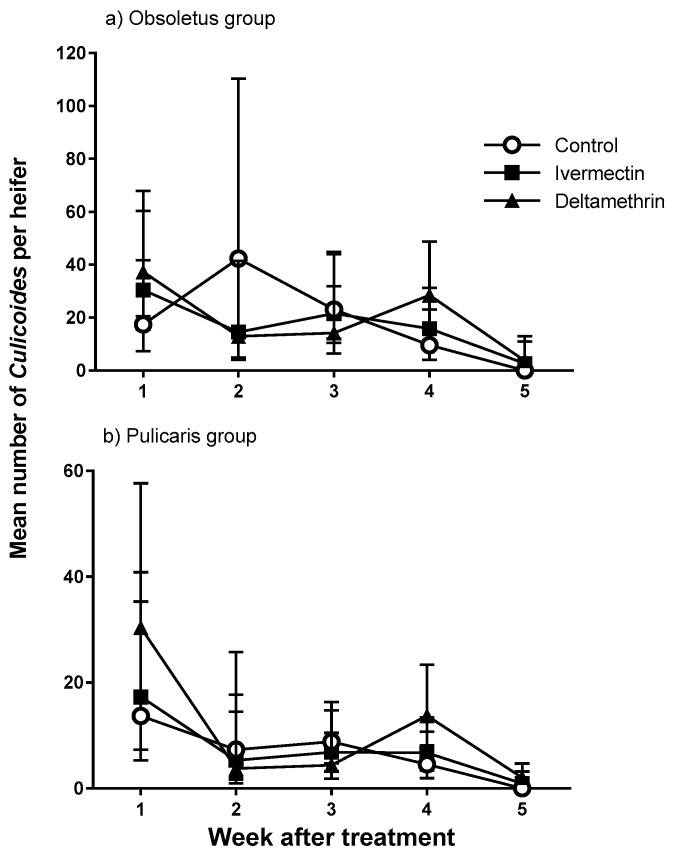
Mean number (±95% confidence intervals) of female *Culicoides* for (**a**) Obsoletus group and (**b**) Pulicaris group captured on white sticky traps, mounted on the backs of Friesian heifers treated with deltamethrin, ivermectin and untreated control (*n* = 5) and released onto open pasture for 24 h, 1–5 weeks after treatment. Data are back-transformed from generalised linear mixed model (GLMM) predictions.

The total number of *Culicoides* caught on sticky traps during this study was 1,889. All were female and belonged to the Obsoletus (67%) or Pulicaris (33%) groups. The numbers of *Culicoides* caught declined as the experiment progressed (Figure 3). However, there was no effect of deltamethrin or ivermectin treatment on the number of *Culicoides* caught on sticky traps (overall *Culicoides* per heifer, back-transformed (b-t) from GLMM predictions with 95% confidence intervals: Obsoletus group − control = 17.03 (11.01–26.32), ivermectin = 18.16 (11.96–27.57), deltamethrin = 19.90 (13.46–29.40), deviance ratio = 0.14, d.f. = 2, 65, *p* = 0.870; Pulicaris group − control = 7.47 (4.44–12.58), ivermectin = 7.79 (4.74–12.81), deltamethrin = 11.23 (7.45–16.94), deviance ratio = 0.92, d.f. = 2, 65, *p* = 0.404; Figure 3).

The proportion of nulliparous midges caught on animal-mounted traps was smaller in the ivermectin treatment than in the control or deltamethrin treatments for both Obsoletus and Pulicaris groups (b-t means, Obsoletus group control = 0.74 (0.64–0.82), ivermectin = 0.55 (0.46–0.65), deltamethrin = 0.68 (0.59–0.76), deviance ratio = 14.73, d.f. = 2, 54, *p* = 0.030; Pulicaris group control = 0.60 (0.42–0.76), ivermectin = 0.32 (0.19–0.50), deltamethrin = 0.61 (0.46–0.74), deviance ratio = 20.50, d.f. = 2, 52, *p* = 0.035).

Two-hundred and eighty-three *Culicoides* were reared from dung collected from the experimental heifers, with these being 106 female and 177 male and predominantly Obsoletus group (95%) and Pulicaris group (5%), with a single midge from the Impunctatus group (<1%). The bucket emergence traps collected 116 midges (41%) and the incubated soil cores 167 (59%). There was no effect of ivermectin or deltamethrin on *Culicoides* emergence at the 5% significance level (Wald statistic = 2.75, d.f. = 2, 53.1, *p* = 0.261); although for the Obsoletus group, *p* was less than 0.1 (b-t means, control = 6.25 (2.48–15.73), ivermectin = 1.76 (0.48–6.39), deltamethrin = 3.27 (1.12–9.52), F = 2.44, d.f. = 2, 68.1, *p* = 0.095).

### 3.2. Trial 2—Culicoides’ Landing and Blood-Feeding on Friesian Heifers Treated with Ivermectin Pour-On

**Figure 4:**
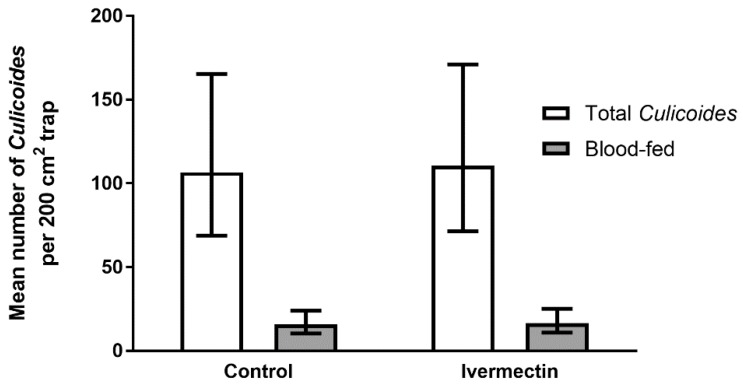
Mean number (±95% confidence intervals) of *Culicoides* and number blood-fed (grey bars) captured on 200 cm^2^ white sticky traps mounted on the backs of Friesian heifers, treated with ivermectin and fenbendazole (control) (*n* = 10) and released onto open pasture for 24 h. Data are back-transformed from GLMM predictions.

A total of 15,766 *Culicoides*, with a maximum of 1,260 caught in a single trap, were collected from the 20 experimental heifers over 24 h. There was no significant effect of ivermectin treatment on the number of *Culicoides* trapped (Wald statistic = 0.01, d.f. = 1, 16.6, *p* = 0.918; Figure 4) or the proportion blood-fed (b-t means, proportion blood-fed, control = 0.14 (0.11–0.19), ivermectin = 0.15 (0.11–0.19), Wald statistic = 0.09, d.f. = 1, 15.2, *p* = 0.774).

### 3.3. Trial 3—Culicoides’ Landing, Blood-Feeding and Mortality on Friesian Heifers Treated with Deltamethrin and Ivermectin Pour-Ons

**Figure 5:**
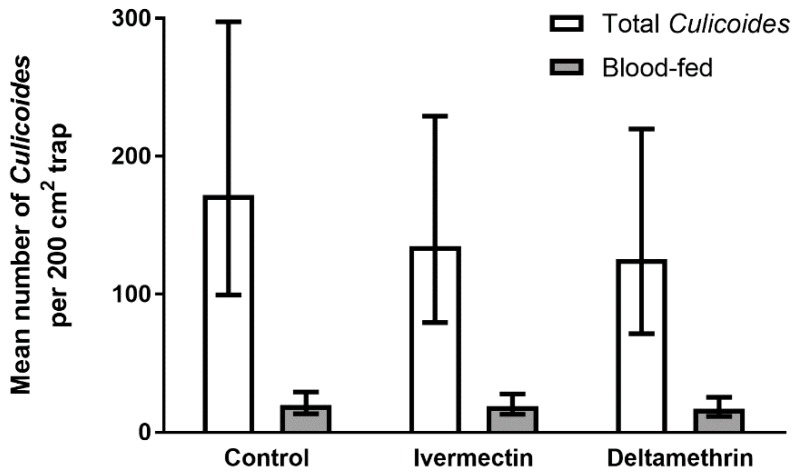
Mean number (±95% confidence intervals) of *Culicoides* and number blood-fed (grey bars) captured on 200 cm^2^ white sticky traps which were mounted on the backs of Friesian heifers treated with ivermectin, deltamethrin or fenbendazole (control) (*n* = 5) and released onto open pasture for four consecutive 24 h periods. Data are back-transformed from GLMM predictions.

A total of 19,720 *Culicoides* (a maximum of 641 on a single trap) were caught on sticky traps on the 15 heifers over the four consecutive 24 h periods. Again, there was no significant effect of ivermectin or deltamethrin on the number of *Culicoides* caught on animal-mounted sticky traps (Wald statistic = 0.95, d.f. = 2, 13.5, *p* = 0.633; Figure 5) or the proportion of midges that had blood-fed (b-t means, proportion blood-fed, control = 0.11 (0.08–0.14), ivermectin = 0.14 (0.11–0.18), deltamethrin = 0.12 (0.10–0.16); Wald statistic = 2.63, d.f. = 2, 11.2, *p* = 0.307).

**Figure 6:**
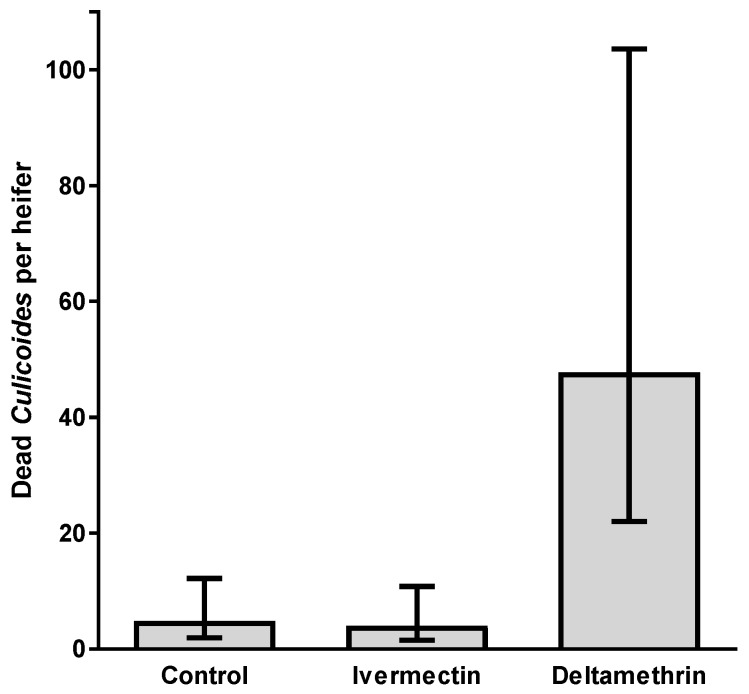
Mean number (±95% confidence intervals) of dead *Culicoides* combed from heifers treated with ivermectin, deltamethrin or fenbendazole (control) (*n* = 5). Data are back-transformed from GLMM predictions.

A total of 737 dead *Culicoides* were combed from the hair of the 15 experimental heifers from samples taken on two consecutive days. There were significantly more *Culicoides* removed from the hair of deltamethrin-treated heifers than those treated with ivermectin or the control heifers (Wald statistic = 19.71, d.f. = 2, 13.1, *p* = 0.002; Figure 6). Due to the dehydrated condition of these midges it was not possible to determine visually if they were blood-fed.

## 4. Discussion

To use insecticides to protect livestock from *Culicoides*–vectored pathogens requires that the relationships between midges alighting on the host, blood-feeding, insecticide-repellence and insecticide-toxicity are understood. In these three trials, there was no effect of ivermectin or deltamethrin on the numbers of *Culicoides* caught on animal-mounted sticky traps, indicating that these treatments did not deter landing. This is perhaps to be expected as the main actions of deltamethrin and ivermectin are toxicity, although there is also some evidence of contact irritancy and repellence to deltamethrin [18,37,38] and ivermectin has reduced the responses of *Culicoides imicola* Kieffer, 1913 to host cues in an olfactometer [39]. Crucially, there was also no difference detected in the proportion of *Culicoides* blood-fed between treatments. The midges caught in this study were fresh blood-fed, which implied that they had fed on the heifers on which they were trapped. After engorgement, haematophagous insects leave the host to digest their blood meal. Therefore, this result indicates that the deltamethrin and ivermectin treatments did not prevent blood-feeding.

Maclachlan and Mayo [5] suggest that insecticides which allow *Culicoides* to blood-feed are not effective in bluetongue control as feeding midges can transmit the virus before dying. Robin et al. [19] found that a topical application of deltamethrin did not prevent blood-feeding on horses but did consider that if such treatments killed midges they could have a role in reducing onward transmission of disease from viraemic horses or suppressing the immediate *Culicoides* population. The combing technique in the present study showed that deltamethrin was killing midges that alighted on the heifers’ upper flanks. Deltamethrin is lipophilic and is disseminated by the natural oil secretions of the coat, with the midges exposed to the insecticide as they crawl through the hair. Deltamethrin is highly toxic to European *C. obsoletus*, with an LD50 of 1.33 × 10^−4^% [40] but despite this, blood-feeding was not prevented. Other studies have found that the insecticide concentration declines from the mid-line of the back to the belly, legs or face [6,15,30,41]. However, in this study, since engorged *Culicoides* were trapped on the middle of the back of the heifers close to the deltamethrin deposition point and surrounded by dead midges in the hair, it is reasonable to assume that these midges were able to blood-feed despite receiving a subsequently lethal dose of insecticide. This differs from the conclusions of earlier studies exposing hair from treated animals to *Culicoides*. These considered that the rapid knockdown of *Culicoides* by deltamethrin would prevent blood-feeding [42,43].

Ivermectin has a different mode of action to deltamethrin. It is highly lipophilic but also systemic, being absorbed through the skin to the subcutaneous fat reserves and bloodstream. It may also bind to the hair and be retained on the skin [31]. There was no evidence of *Culicoides* mortality in this study but this cannot be discounted as ivermectin does not have the same knockdown effects as deltamethrin and midges may fly from the animal before dying. Furthermore, sub-lethal effects may have a role in suppressing disease transmission through reduced lifespan, fecundity, dispersal or altered vector-pathogen interactions. A good example of this is that ivermectin reduced the bluetongue (BTV-17) infection rate of *C. sonorensis* by 40% with a 29% reduction in dissemination of the virus from the midges’ bodies to the heads [23]. The only significant effect of ivermectin on *Culicoides* in this study was a reduction in the proportion of nulliparous midges caught. The reasons for this are unknown. Different physiological states can influence how vectors respond to host cues. It is possible that nulliparous females are more sensitive to stimuli than older parous females [44] and are deterred by subtle changes in the host olfactory profile brought about by ivermectin treatment [39].

Ivermectin is excreted in the animals’ dung and can have negative effects on dung-dwelling fauna [45,46,47], including nuisance and biting fly larvae [48,49]. In this study, the numbers of *Culicoides* that emerged from the dung samples was small. There was a tentative suggestion of a negative effect of ivermectin on emergence of the Obsoletus group, the main vector risk group, but this was not significant at the 5% level (*p* = 0.095). Nevertheless, ivermectin excreted in the dung is not considered a viable widespread strategy to reduce *Culicoides* population levels due to the adverse effect on beneficial non-target invertebrates and also the availability of other larval breeding sites for *Culicoides*. Deltamethrin may also have residual insecticidal activity in dung [50,51].

The technique of using sticky traps to provide a standardised method of assessing *Culicoides* landing on experimental animals generally worked well. The numbers of *Culicoides* caught on traps were variable but in the second and third trials in September, they were substantial. In the third trial, a mean control of 172 (maximum of 1260) *Culicoides* were caught per 200 cm^2^ trap over a 24 h period. It is unknown to what extent these traps were attracting *Culicoides* to land on them compared to the surrounding animal’s skin. However, if such figures are extrapolated to the surface area of the heifers, and allowing for preferential feeding on different areas of the body, then animals in this location face a biting intensity of tens of thousands of *Culicoides* per night. One disadvantage of sticky traps is that specimens are distorted and difficult to identify to species level. Light trap catches at this location were mostly Obsoletus group [52], a subsample of which were subsequently identified to species level with *C. obsoletus* s.l./*scoticus*, *Culicoides lupicaris* and *Culicoides dewulfi* predominant (Bruno Mathieu and Thomas Balenghien, pers. comm.) Although UV light trapping may overestimate the biting rates of *C. obsoletus*, they nonetheless give a reasonable indication of the species involved [53].

## 5. Conclusions

The main result from this study is that neither deltamethrin nor ivermectin prevented blood-feeding by *Culicoides* midges on cattle in a field situation. The implications of this are that these treatments do not have a protective effect for individual animals but could prevent onward transmission of pathogens via post-feeding mortality of the vector. This would certainly seem to be the case for deltamethrin, where large numbers of dead *Culicoides* were collected on the upper flanks of the animals. For ivermectin, it was not possible to assess the mortality of affected *Culicoides* as they most likely died off the animal. Therefore, at the present time, in cases of suspect imported livestock, which represent the most significant risk of bluetongue incursion into Ireland, application of deltamethrin as a pour-on or spray treatment applied to the quarantine animals would be advocated to reduce the risk of onward transmission. This should be combined with spraying of the housing using a residual insecticide and where possible screening of the entrances. Any native livestock on the farm should be situated as far as possible from the quarantine housing, although it should be considered that *Culicoides* can disperse up to 5 km within a few days [54].

Although the sticky trap technique worked well, the position of the traps was restricted to the mid-back where the heifers could not lick or swat the traps with their tails. The coverage provided by pour-on treatments diminishes with distance from the application site, so in a future study it would be valuable to assess *Culicoides* mortality in distal areas with maximal midge feeding, such as the underbelly [26], possibly using a sticky substance applied directly to the hair rather than sticky plastic plates.

## Figures and Tables

**Table 1:** Timing of treatments (days after application of ivermectin or deltamethrin) and sampling of heifers for *Culicoides* using sticky traps and combing in two consecutive trials in a Northern Irish pasture. The ivermectin-treated animals from trial 2 were also sampled in trial 3.

Trial Number	Date	Days after Ivermectin Treatment	Days after Deltamethrin Treatment	Activity
Trial 2	28 Aug	0	-	**Ivermectin applied**
	2 Sep	5	-	Sticky traps attached
	3 Sep	6	-	Sticky traps collected and attached
	4 Sep	7	-	Sticky traps collected
Trial 3	4 Sep	7	0	**Deltamethrin applied**
	8 Sep	11	4	Sticky traps attached
	9 Sep	12	5	Sticky traps collected and attached
	10 Sep	13	6	Sticky traps collected and attached
				Flanks combed
	11 Sep	14	7	Sticky traps collected and attached
				Flanks combed
	12 Sep	15	8	Sticky traps collected
				Flanks combed
